# German physicians' expectations of healthcare management companies: An exploratory study

**DOI:** 10.1002/hsr2.866

**Published:** 2022-10-29

**Authors:** Dirk Oberschachtsiek, Andree Ehlert

**Affiliations:** ^1^ Harz University of Applied Sciences Wernigerode Germany

**Keywords:** cooperation, coordination, healthcare management company, integrated care, managed care

## Abstract

**Background and aims:**

Even 20 years after the introduction of managed care (MC) in Germany, many physicians are skeptical of the concept, hindering its acceptance.

**Methods:**

Based on multivariate statistical methods this exploratory study examines how so‐called management companies, that is, administrative service providers within MC contracts, can increase physicians' acceptance of MC by offering, for example, day‐to‐day coordination and administrative tasks.

**Results:**

As a main empirical result, we find support for this hypothesis, that is, that certain physicians evaluate their MC participation according to its prospective administrative support. Based on this, up to four clusters of physicians can be statistically identified in terms of their preferences regarding MC.

**Conclusion:**

As a policy recommendation, we derive from our results that a future focus on the administrative support components of MC is essential to attract certain physician groups to participate in MC.

## 1 INTRODUCTION

The concept of managed care (MC) has received a great deal of attention internationally in recent decades as a means to improve collaboration between health care providers. However, the exact meaning and definition of MC varies greatly from country to country as the design of MC is also determined by the requirements of the respective health care systems. In Germany, for example, a very open interpretation of MC is often applied. In particular, all steps that could lead to an improvement in the cooperation of individual health professionals as compared to standard care are often termed “MC.”

However, the implementation of such MC concepts in Germany since the early 2000s has fallen short of expectations with an insufficient number of participants on the part of the insured^1^ and, at the same time, a lack of willingness on the part of health care providers to participate in MC. A variety of reasons for this have been extensively discussed in the literature. For example, it has been found that German physicians seem to be rather reluctant to accept performance‐based payment systems which are often found under MC.^2^ In addition, physicians seem to have strong concerns about MC contract design, which tends to be highly standardized by insurers, see.^3^ There is also a general fear of a loss of physician autonomy under MC contracts as health care providers will give up at least some of their professional autonomy in favor of control by payers.^4^ On the other hand, studies also suggest that certain aspects of MC, such as quality circles or shared decision making in themselves, have high acceptance levels within the medical profession.^5^, ^6^, ^7^ This shows that there does seem to be a general potential among physicians for further expansion of MC in Germany.

In fact, there are only few publications that address expectations of how MC should be organized and what elements MC should include from the perspective of potentially involved actors (e.g.,^8^; for a general discussion of the extant literature see Section 2.2 below). To the best of our knowledge, there is no published paper to date on our specific research question, namely, what expectations health care providers have about the possibility of transferring management functions (e.g., ongoing organizational practice and billing tasks) to a so‐called management company operating as an organizational or service unit within an MC contract. Consequently, this study asks where suitable areas of responsibility for management companies could lie from the perspective of healthcare service providers. Note that to analyze this question, we take a very broad view of MC^9^, ^10^ in line with German legal requirements (see § 140a SGB V, German Social Code, Book V). This means that in addition to its traditional focus on collaborative health care, we also emphasize the organizational and management aspect as a valid element of MC.

To this end, we ask what expectations physicians have specifically of the management component (i.e., the administrative unit) under an MC contract providing a precise description of the management task involved in coordinated networking from the physicians' point of view. A specific questionnaire was developed for this purpose. The attractiveness of administrative workload reduction as a possible component of MC contracts is then examined in an empirical survey from the perspective of the medical profession.

Our findings provide important insights for designing MC contracts to be more incentive‐compatible for physicians in the future. This would involve marketing management companies to physicians as a type of service provider that offers tangible benefits. Examples include administrative support, financing, IT networking of physicians, or risk pooling.^11^

Quantitative empirical research on this question is hardly available for Germany so far. Some findings on perceived importance and expectations of content‐related MC aspects have been discussed, for example, by Refs. 6, 7, 10, 12, 13 Outside of the German healthcare system, there is some literature that addresses specific facets of MC management components.^14^ Further studies in this context include selected aspects on data and information exchange^8^, ^15^ or the design of controlling aspects.^16^, ^17^, ^18^, ^19^

Based on a physician survey our paper applies an empirical factor and cluster analyses to identify major components of physicians' expectations from a healthcare management company associated with a MC‐contract. The analysis also allows us to find several potentially latent groups of physicians based on these factors. From a health policy perspective, these findings can serve for target group‐specific communication with the medical profession and thus increase the attractiveness of MC participation. The data set is based on a structured telephone survey of 500 physicians with own practice in Northern Germany.

The remainder of the paper is organized as follows. Section 2 provides background information on the role of management companies in Germany and a literature review. Section 3 describes the data collection and the methodology used. The empirical results are presented in Section 4. Section 5 provides a discussion. Section 6 concludes.

## 2 RESEARCH FRAMEWORK

### 2.1 The role of management companies in German MC

Before going into more detail about the existing literature and the gap therein that leads to our research question, we will briefly discuss the specific understanding of MC in Germany compared to other countries. This is all the more important because our research question is also directly related to the relatively broad definition of MC in Germany. The latter allows the establishment of so‐called management companies within MC as a German peculiarity (see § 140a SGB V). Management companies may provide unspecified administrative and organizational services to MC contract partners.

In the United States, for example, MC has been introduced since the 1970s mainly in terms of Health Maintenance (HMO) and Preferred Provider Organizations (PPO). While HMOs focus on the integration of insurance and provider activities within a single organization, PPOs manage the utilization of health care services within a provider network.

In German health care, similar attempts have been made to establish a stronger sectoral integration with the Social Health Insurance Reorganization Acts starting in 1997 (GKV‐Neuordnungsgesetze). In particular, the subsequent reform attempts based on §140a (integrated/special Care, IV), §137 f (disease management programs, DMP) and §§63‐65 (model projects), SGB V, reflect a strengthening of care options in the sense of MC. The basic idea behind these reform approaches involves selective contracts between individual service providers, such as IV or DMP. For example, one aspect of these reform approaches is that utilization of services is controlled by financial or qualitative incentives (e.g., limiting the patient population to participants, continuing education requirements for physicians, referral only to network physicians, etc.).

The function of the management company as defined in §140a SGB V plays a special role in the design of these selective contracts. In essence, the legislator is concerned to bring business expertise and coordination into selective care via management companies, thereby ensuring their economic survival in a competitive environment.^20^ A broad interpretation of the term “management company” is obviously intended by the legislator. Since health care providers and insurers generally have little experience with MC in Germany, a division of labor with respect to management tasks seems reasonable. However, this is accompanied by an inherent conflict of interests between adequate service provision and cost‐efficient management.

The pure service provider function may be contrasted with a more strategic function of the healthcare management companies. This view discusses the latter primarily as a central component of systemic innovation for healthcare systems. This involves a patient‐oriented reorganization of the individual delivery of healthcare services. Here, management companies provide additional services such as patient coaches and appointment management. They may also serve as an additional intermediary between service providers and health insurers. In this way, budget control and contract management can be complemented by population and information pooling. An example of this more strategic role for management companies is provided by OptiMedis AG.^21^ In the following section, we will briefly discuss that the preferred role of management companies from the physicians' perspective is a promising research question that has not yet been addressed widely in the literature to date.

### 2.2 Literature review and research approach

First, we will provide a rough framework for the different understandings of MC in the extant literature. This is followed by a more specific literature review around the definition chosen here to guide our research question.

To begin with, it is helpful for our discussion to roughly distinguish three ways of defining MC in the literature: First, as a focus on the precise description of institutional design such as HMO, PPO, point of service, or exclusive provider organizations, see for example Ref. 22 Second, from a process perspective as a specific arrangement of functions, services, or instruments.^14^, ^16^, ^23^ And third, representing the broadest definition (which is also used in this study), MC can be understood as a general arrangement for the division of labor in health care, possibly across different sectors. From the point of view of the physicians involved, MC can thus essentially be seen as an approach in which certain management functions and tools are offered in support of care.^24^ This third approach now also makes it possible to understand the management companies discussed in Section 2.1 as a component of MC.

In the context of the latter (broad) definition, the conditions for success and acceptance of MC are well analyzed in the literature. For example, it has been emphasized that knowledge of the capabilities of actors involved as well as an organized contact structure between actors can be important aspects for the success of MC.^23^, ^25^ Communication and respect are further core elements emphasized in these studies for a functioning division of labor in MC arrangements. Further papers also discuss the particular importance of incentive and control structures in MC systems based on division of labor.^26^, ^27^, ^28^, ^29^ The papers of Cakici and Mills, Eigner and Hamper^30^, ^31^ also refer to special acceptance conditions when specific technologies are integrated into the structures based on the division of labor.

A study by Deom et al.^10^ also follows the third definition where MC is seen as a kind of mixed bundle that uses different instruments to coordinate care. In their paper, the individual instruments (treatment guidelines, referral models, networks, second opinion model, fixed payment, fee‐for‐service, treatment evaluations, and utilization review) were to be evaluated by 1546 physicians in Geneva, Switzerland, based on different evaluation criteria: quality of care, cost control, autonomy of care, and patient relations. Most of the listed tools were considered helpful for cost control by the physicians. But, at the same time, they rated the tools as rather moderate to negative in practical application (more precisely: in terms of ensuring treatment autonomy and supporting patient relations).

Similarly Rischatsch and Zweifel,^7^ study the monetary compensation required by physicians to accept individual MC components. The study is based on decisions about the choice of hypothetical MC service bundles using written surveys of 1088 practicing physicians. Eight typical MC features are used to construct hypothetical situations (e.g., shared decision making or quality circles). For most MC components, they find that monetary compensation is required, whereas no compensation is only required for up to six quality circle appointments and for seeking second opinions. The results underscore that physicians, in general, are skeptical of interference in medical decisions through MC contracts.

Based on 150 network participants,^6^ examines the importance physicians attach to individual MC aspects and their expectations of MC contracts. The study shows that network members value above all the possibility of selective contracts as well as the possibility of improved teamwork and opportunities to improve the quality of treatment. Another important aspect from the perspective of participating physicians is the fact that they associate MC contracts with an option to secure their economic future. Network participants primarily expect better treatment quality, followed by uniform treatment standards, modernization impulses, and positive income effects.

The overview discussion of the literature above reveals a certain research gap in three respects, which our paper addresses. First, the importance of different management components associated with MC is generally omitted in favor of, for example, the medical and monetary aspects of MC. Second, most studies that address management aspects of MC are limited to qualitative approaches that, unlike our paper, do not allow for multivariate analyses, see e.g. Weinmayr et al.^25^ Finally, none of the previous studies focuses on the heterogeneity of physicians in terms of their expectations of MC. However, it is the analysis of this physician heterogeneity, which we will discuss in Sections 4 to 6, that will enable policymakers to advance MC adoption in a more target‐group‐specific manner than has been the case to date.

## 3 MATERIALS AND METHODS

### 3.1 Sample and data

The data we use is based on a CATI (Computer‐assisted telephone interviewing) survey of 504 physicians in private practice in Northern Germany, that is, Lower Saxony, Schleswig‐Holstein, Hamburg, and Bremen. The region includes a mix of rural and urban areas with three major metropolitan areas. For a critical discussion of this method, see Refs. 32, 33 The survey was conducted between August and September 2014 based on a population of approximately 6000 telephone register data. Twenty‐seven professional interviewers trained for the project were employed and conducted the telephone survey between 8:00 a.m. and 8:00 p.m. on all days of the week. On average, seven contacts were required for a successful telephone interview, which lasted 15–16 min on average. Participation was refused in 3469 cases. Other dropouts were related to “not reached” (*n* = 808), “answering machine” (*n* = 950), “appointment but no one reached” (*n* = 188), “repeatedly busy” (*n* = 26), and “dropout without continuation” (*n* = 14). Regarding the participation rate, it should be considered that no compensation was granted. The limited accessibility of physicians for an interview is also reflected in the relatively high median of six contact attempts for a successful interview.

The distribution of the characteristics gender, age, specialist qualification and single practice largely corresponds to the structural characteristics of all physicians in private practice in Germany.^34^ About 68% of the surveyed participants were specialists, the rest were family physicians.

### 3.2 Measures and variables

The focus of the survey is the cooperation and networking behavior between physicians in private practice with other health care providers. All physicians were asked whether they were already involved in certain forms of cooperation and MC. In addition, general structural information on practice and professional orientation was collected. The questionnaire, which was developed specifically for the project itself, considered the results of a qualitative preliminary study. The questionnaire was finally validated with a pretest (*n* = 40) under practical conditions.

The sample was split for more in‐depth focus surveys. In one of these focus surveys, a total of 95 physicians were asked which services they would like to see management companies take over when it comes to collaborating with other health care providers within MC. Respondents were given a list of possible tasks to evaluate. Specifically, the survey stated, "Imagine you decide to collaborate with others. A coordination office is to be established to support this endeavor. This coordination office can now have different focal points of work. How important do you consider the following tasks on a scale from 1 = completely unimportant to 5 = very important?" The following services were listed:

- It helps to solve the documentation tasks better
- It implements case management
- It coordinates appointments with specialist colleagues for faster diagnoses
- It advises and supports patients, for example, in questions of their treatment organization
- It organizes specialist circles and case discussions
- It helps to avert problem cases and recourse risks
- It is responsible for administrative tasks.

### 3.3 Data analysis procedure

Our data analysis procedure can be divided into four steps. In the first step, we perform summary descriptive analyses of the responses in tabular form (including mean and standard deviation, SD). In the second and third steps, we perform a multivariate analysis of the responses to find latent structures that help us to characterize groups of physicians that are homogeneous in terms of their expectations as to the management component of MC. Steps 2 and 3 have been carried out with the Stata 16 software.^35^ A factor analysis (Step 2) to search for latent characteristics is followed by a cluster analysis (Step 3) to identify potential groups of physicians with similar expectations as to management companies. The cluster analysis first uses the results of an agglomerative‐hierarchical procedure to identify the optimal number of existing groups in the data. It then applies a kmeans analysis to obtain a cluster‐assignment of the investigated physicians. In a fourth step, these clusters are characterized for ease of interpretation using summary data for the practice manager (physician) and the practice itself.

## 4 RESULTS

### 4.1 Descriptive analysis

Table 1 provides a descriptive overview of the healthcare providers included in the sample. It also shows the subsample of physicians who completed the focus survey section “Expectations of management companies” and compares these with the entire sample (*t*‐statistics, see last column, none of which are statistically significant at the usual levels).

**Table 1:** Basic sample characteristics

	Total sample		Subsample without focus survey		Subsample with focus survey		Mean difference
	*n*	Mean (SD)	*n*	Mean (SD)	*n*	Mean (SD)	|*t*‐statistic|
General practitioner (1 = yes, 0 = no)	504	0.31 (0.02)	411	0.32 (0.02)	93	0.27 (0.05)	0.98
Number of doctors in practice	502	1.32 (0.05)	410	1.32 (0.05)	92	1.30 (0.09)	0.19
Practice in rural to small town area (1 = yes, 0 = no)	504	0.45 (0.02)	411	0.46 (0.02)	93	0.41 (0.05)	0.85
Age of practice leader (years)	481	56.60 (0.40)	394	56.44 (0.44)	87	57.32 (0.94)	0.85
Share of private patients (%)	486	21.00 (1.19)	397	21.08 (1.33)	89	20.63 (2.60)	0.15
Number of cooperation partners	504	3.87 (0.09)	411	3.91 (0.10)	93	3.65 (0.19)	1.17

About 31% of the physicians are general practitioners. In terms of full‐time staff, approx. 1.32 physicians are employed in the practices, with one or more part‐time staff in 35 cases. Nearly 90% of these involve a maximum of two part‐time employees working in the practice. Nearly half of the practices are in a rural to small‐town environment. The physicians are involved in an average of nearly four cooperation forms (i.e., the sum of the forms of cooperation in which the surveyed physicians confirmed their participation). Information on the age of the practice leadership and the proportion of private patients was only provided by 486 and 481 physicians, respectively. This could be due to a higher degree of uncertainty if the respondent is not a member of the practice management. The average age of the practice head is ~57 years. In addition, the proportion of private patients is around 21%. Table 1 also shows that the physicians who participated in the special survey are not statistically different (95% level) from the group that did not receive the focus questions. The following analyses are based on the subsample who received the focus questions.

Table 2 shows how important the physicians rated the individual possible tasks and functions of management companies (sorted in descending order of importance, mean values).

**Table 2:** Physician ranking of possible functions expected from management companies

	*N*	Mean	SD	Min	Max
Administration	94	3.37	1.40	1	5
Documentation	95	3.24	1.48	1	5
Recourse defense	94	3.21	1.45	1	5
Specialist circles and meetings	95	3.18	1.34	1	5
Appointment coordination	95	3.11	1.43	1	5
Case management	93	2.99	1.43	1	5
Treatment organization	94	2.94	1.38	1	5

Table 2 indicates that the surveyed physicians do not see any major differences in the importance of individual possible areas of activity for healthcare management companies. By ranking, it can only be roughly seen that services such as the implementation of administrative tasks, documentation, or defense against recourse have slightly higher approval ratings in relative terms. At the same time, case management and treatment organization are rated as comparatively less important by physicians. Further comments reported by the physicians in an open question include additional tasks for management companies such as the implementation of purchasing discounts, financing advantages, training, quality management, information bundling, and network organization at the site.

### 4.2 Dimensions of expectations

The starting point is a principal factor analysis^36^ based on the seven characteristics used to determine the management company's “desired focus of work,” see Section 3.2. The measure of sampling adequacy according to Kaiser–Meyer–Olkin (with a value of 0.87 for all included characteristics) confirms a latent data structure.^37^ The Bartlett test of sphericity also confirms the suitability of the data for factor analysis. It turns out that based on the Kaiser criterion, a one‐factor solution is the preferred data representation.

A complementary principal‐component factors solution^36^ shows that considering a second factor provides an additional variance coverage of 11.47% (first factor: 61.42%). Moreover, the information score in the model comparison is highest for the two‐factor solution (lowest AIC and BIC values). Therefore, the two‐factor solution is used throughout the rest of the analysis.

The factor table obtained after varimax rotation (see Table A1, factor loadings below 0.3 are suppressed) shows that Factor 1 captures the expectations “documentation” (factor loading 0.838), “appointment coordination” (factor loading 0.751) and “administration” (factor loading 0.727). The interpretation is that the factor represents a bundle of expectations in the direction of “administrative process support”. Note that with a Cronbach's alpha of 0.874 we obtain sufficient support for our general interpretation of this factor.

Factor 2, by contrast, can be interpreted as (a bundle of) expectations for “extended treatment support” (with factor loadings of 0.695 on “treatment organization” and 0.501 on “specialist circles”). With respect to its internal consistency, we find an acceptable Cronbach's alpha of 0.671 (where adding the variable “recourse defense” to the factor would produce a Cronbach's alpha of 0.740).

As a robustness check we allowed both factors to be interrelated (using Stata's rotation procedure “oblimin oblique”). Then, the variables “specialist circles” and “case management” have considerably higher relevance for the second factor. The relevance of the core characteristics identified in orthogonal rotation also increases. Overall, the representation of the factors in terms of content remains the same for different rotation procedures. The robustness of the results is also underlined by the fact that all common rotation methods support a two‐factor solution.

### 4.3 Physician groups with different expectations

**Figure 1:**
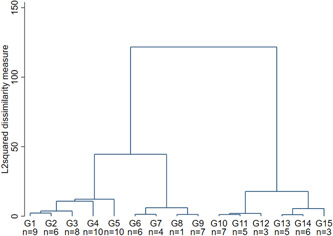
Dendrogram based on two‐factor solution from Section 4.2

The starting point for the cluster analysis is an agglomerative‐hierarchical approach. The results of the procedure are summarized in the dendrogram in Figure 1. As can be seen, the inequality increases especially in the penultimate merger stage, which indicates a four‐cluster solution. Against this background, the clusters labeled G1 to G5, G6 to G9, G10 to G12, and G13 to G15 in the figure appear to represent similar groups. However, the degree of dissimilarity only increases substantially in the last stage, so that a three‐cluster solution could also be considered (G1 to G5, G6 to G9, and G10 to G15). Note that a two‐cluster solution does not seem to be a suitable solution according to the elbow criterion.

In addition, Table A2 shows statistical indicators that can be used to identify the optimal number of clusters based on the Calinski/Harabasz pseudo‐F statistic and based on the Duda/Hart index.^38^ High values represent distinct clusters. Both the Calinski/Harabasz‐pseudo‐F statistic and the Duda/Art statistic support the above argumentation regarding a three‐ to four‐cluster solution in the data.

**Figure 2:**
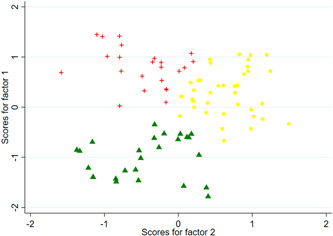
Factor scores for the three‐cluster solution

**Figure 3:**
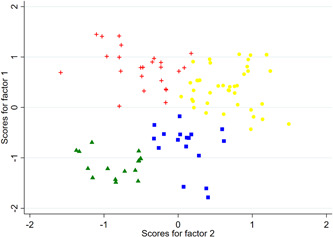
Factor scores for the four‐cluster solution

For the second step, the procedure according to the kmeans method (Euclidian distance) for the three‐ and four‐cluster solution is shown in Figures 2 and 3. Table 3 additionally provides the average factor loadings per cluster (upper part of the table) and the scores of the variables included in the factor analysis (lower part of the table). Regarding the three‐cluster solution, we find cluster 3/1 to be a group with predominantly negative factor loadings on both extracted factors. In terms of content, this group thus has fundamentally low approval ratings for management services in the context of cooperation with other service providers and can best be described as a “rejection group.” Cluster 3/2, on the other hand, contains observations with negative factor loadings for factor 2, while at the same time having positive factor loadings for factor 1. Hence, this cluster corresponds to a group of physicians with high agreement on administrative services in MC, but at the same time with lower agreement on professional‐oriented support services (represented by factor 1). It could be described as a “management group.” Finally, the third cluster 3/3 includes observations with mixed positive and negative factor loadings with respect to both factors. Table 3 shows that in cluster 3/3 the support components case management, treatment organization, and recourse defense have the highest agreement values. This stands in contrast to cluster 3/2 where administration, documentation, and appointment coordination show high agreement, while treatment organization and case management exhibit low to moderate agreement. As expected, Table 3 confirms that in cluster 3/1 the approval ratings for all potential support components of a management company are low.

**Table 3:** Comparison of mean factor loadings (upper two rows) and answer scores (lower part) for the respective cluster solutions

	Three‐cluster solution			Four‐cluster solution			
	Cl 3/3 (*n* = 39)	Cl 3/2 (*n* = 23)	Cl 3/1 (*n* = 30)	Cl 4/2 (*n* = 38)	Cl 4/4 (*n* = 23)	Cl 4/1 (*n* = 18)	Cl 4/3 (*n* = 16)
Factor 1	0.33 (0.077)	0.79 (0.082)	−1.06 (0.074)	0.41 (0.071)	0.79 (0.082)	−1.21 (0.59)	−0.77 (0.117)
Factor 2	0.69 (0.610)	−0.47 (0.095)	−0.536 (0.106)	0.69 (0.062)	−0.51 (0.090)	−0.95 (0.073)	0.11 (0.074)
Documentation	3.92	4.43	1.48	4.00	4.41	1.31	2.00
Case management	4.08	3.04	1.59	4.19	2.95	1.19	2.25
Appointment coordination	3.68	4.00	1.62	3.83	4.00	1.12	2.19
Treatment organization	4.10	2.17	1.96	4.11	2.13	1.19	3.06
Specialist circles and meetings	4.08	3.13	2.03	4.05	3.13	1.62	2.81
Recourse defense	3.97	3.35	2.10	4.05	3.32	1.12	3.25
Administration	3.95	4.22	1.86	4.08	4.23	1.31	2.50

*Note*: Values in parenthesis indicate standard errors of the mean.

The four‐cluster solution further differentiates the previous group assignments. The division of the clusters can be described by the proposed quadrant scheme relating to the factor loadings along the two extracted factors, see Figure 3. In view of the factor scores, cluster 4/1 can be described as “skeptics of management services,” that is, both technical and administrative support services have low approval ratings in this group. Cluster 4/2 is the exact opposite. Cluster 4/3 is a “treatment‐only advocate group” which typically contains physicians who have higher agreement with professional support and lower agreement with administrative support. Cluster 4/4, on the other hand, sees less need for specialist support, but is more in favor of administrative services, that is, a “management group.” As with the three‐cluster solution, this picture is also reflected in the average approval ratings for the support components under consideration (see Table 3, bottom section).

A multivariate analysis of variance also confirms statistically significant differences at the 99% level between the groups in the three‐ and four‐cluster solutions. However, the test requirements regarding interval scaling, normal distribution of the factor values and equal distribution in the groups are only partially met. Overall, however, the complementary tests favor the four‐cluster over the three‐cluster solution.

### 4.4 Cluster typology

To further characterize the clusters, Table 4 provides an overview of descriptive statistics for selected characteristics of the physicians included in the study based on the cluster‐assignment of the kmeans procedure. In contrast to Table 3, practice characteristics and some practice manager data are addressed here to obtain a meaningful typology of the physicians included in the clusters.

**Table 4:** Descriptive statistics of the cluster solutions

	Three‐cluster solution			Four‐cluster solution			
	Supporters	Only admin	Rejectors	Supporters	Only admin.	Rejectors	Professional
	Cl 3/3 (*n* = 39)	Cl 3/2 (*n* = 23)	Cl 3/1 (*n* = 30)	Cl 4/2 (*n* = 38)	Cl 4/4 (*n* = 23)	Cl 4/1 (*n* = 18)	Cl 4/3 (*n* = 16)
Practice in rural to small town area	0.50	0.39	0.31	0.53	0.36	0.31	0.31
Age of practice leader	57.83	55.73	58.26	58.34	55.24	58.47	57.29
Number of doctors in practice	1.21	1.30	1.46	1.31	1.23	1.13	1.63
Number of cooperation partners	3.50	4.39	3.21	3.64	4.36	3.06	3.19

In the following description, the two identified cluster solutions are taken up together. For example, the two clusters with more global approval (cluster 3/3 and cluster 4/2) are characterized by a significantly higher proportion of practices in rural areas. In contrast, the two clusters that focus their approval of support by management companies particularly on administration, documentation, and appointment coordination (cluster 3/3 and cluster 4/3) are on average somewhat younger and have a comparatively high number of collaborative involvements. On the other hand, the two clusters that are fundamentally critical of support by management companies (cluster 3/1 and cluster 4/1) tend to be larger (more physicians employed) and they have rather few collaborative involvements. Interestingly, however, an almost similar picture is also found for the additional cluster of the four‐cluster solution (cluster with comparatively higher agreement for professional support).

## 5 DISCUSSION

The starting point of our analysis was the puzzle that an innovation like MC, which is generally viewed positively from a health economic and medical perspective, has spread only very slowly in Germany over the past 20 years. However, critical discussions regarding a successful implementation of MC can already be found in the early literature.^24^, ^39^, ^40^ Schulz et al.,^24^ for example, argue that in addition to the good reasons for increasing the use of management approaches in health care, there may also be reservations on the part of physicians with regard to their autonomy of care and adherence to quality standards. Feldman et al.^39^ confirm these hypotheses based on survey data of physicians involved in MC settings in Pennsylvania. Similar results are provided by Hadley et al.^40^ for physicians in US metropolitan areas involved in HMO models. Deom et al.^10^ also confirm general reservations among physicians about MC approaches.

Our strategy for identifying concrete starting points to reduce physicians' reservations about MC was to begin with the broadest possible definition of MC (according to the legal framework in Germany). Here, MC is seen as a concept to strengthen the division of labor in the health care system, see Section 2.2. We assumed that the involvement of a management company plays a central role to take over the (“annoying”) administrative part of the daily practice work. A similar approach is taken by Bax et al. and Deom et al.^10^, ^41^ for example, describe MC in general terms as an arrangement of various components that enable the control and management of cross‐sectoral care services.

In this study, we obtain for the first time quantitative feedback on preferences about the desired MC arrangements among physicians. These results are based on the evaluation of possible service bundles offered by management companies in the context of MC. In doing so, our work follows the basic approach of Deom et al.^10^ Note that, unlike our study, they do not consider specific management functions, but rather bundles of services such as the use of guidelines, organized physician exchange rounds, pay‐for‐performance, and selective contracting. Further aspects that are generally discussed outside the medical field as potential success factors in models based on division of labor (including values, respect, knowledge of the capabilities of participating actors, communications, or incentive models) remain unconsidered in our study (from our point of view also as a space for future research). However, these dimensions are taken up, for example, in the analyses of Refs. 23, 25, 26, 27, 42

A key finding of our study is that the empirical analysis reveals two factors regarding physicians' preferences for MC. The first can be described as “administrative process support” and the second as “extended treatment support.” Note that both dimensions were discussed early in the context of MC.^24^ Treatment support (e.g., in the form of case conferences, informal networks, etc.) actually forms the core component of many MC contracts in Germany. However, as to the other factor of “administrative process support” there has only been anecdotal evidence so far about the tense administrative situation of many medical practices (“physicians are frustrated by the administrative burden”).

Janus et al. and Stone^43^, ^44^ provide some indications of the potential relevance of this. Stone, for example, finds that management skills are rated as extremely relevant by physicians themselves, but that such competencies have so far been given little consideration in training. Janus et al.^43^ show for Germany that administrative aspects have a strong negative influence on job satisfaction.

Accordingly, there is likely to be a strong demand to decouple such management aspects from care processes. The indications of Schulz et al.^24^ already point in a similar direction, namely that a focus on improved management processes in health care can also lead to substantial improvement in cooperation, process control and cost control. Thus, in essence, the two factors we found empirically reflect for the first time what has already been reported as two central aspects of the pros and cons of more management (see above).

In addition to providing the first quantitative assessment and multivariate analysis of such outcomes, our study also goes one step further by providing a data‐based typology of physicians with respect to these two factors. In this context, the assignment of physicians to one of the identified clusters reflects a disclosure of their preference, see for example, Zonneveld et al.^45^ A similar finding related to the existence of different subtypes of physicians has to our knowledge previously only been reported by Sohn et al.^46^ in the context network involvement of physicians. In our analysis, we find that physicians can be classified in up to four groups when focusing on their expectations of management services within MC. Two of these four groups reflect a clear positioning (i.e., one group with preference for much process support as well as much treatment support and one group that rejects both aspects) and two mixed groups, each preferring only one of the two aspects. With respect to previous research as reported in Section 2, our findings also support the potentially high relevance of setting up incentive systems^27^, ^42^ to motivate physicians to participate in MC.

## 6 CONCLUSION

The practical relevance of our (cluster‐analytic) results lies particularly in the possibility of creating target‐group‐specific policy measures to promote MC participation. From a health economic perspective, the two mixed groups of physicians (preferring either process or treatment support) are particularly interesting for setting policy incentives for MC participation. This is because fundamental support for the idea of MC already exists in both cases (even if only in one dimension each). Building on this, new low‐threshold MC services could be introduced to these physicians with little effort, focusing either on administrative process support only or on treatment support. To the best of our knowledge, the first type of MC contracts (administrative process support) has existed only marginally in Germany to date or is not even perceived by the public. These basic MC offerings could then provide a broadly accepted starting point for modular MC add‐ons (if needed by physicians) around the respective missing options (e.g., in‐depth cooperation and professional networking).

Concrete measures mentioned in our survey included the following policy approaches as examples for the potential future design of MC services. In addition to documentation support and appointment coordination, negotiation support for discounts and the assumption of administrative tasks, documentation tasks or recourse defense by a management company were also mentioned. However, the transfer of strategic practice management tasks to such management companies also appeared desirable, for example, the negotiation of purchasing discounts and financing advantages or continuing training management and the development of supply networks.

However, our multivariate analyses also revealed that, from a health policy perspective, we still have a very different starting situation in Germany with regard to MC than in the United States, for example. In Germany, there is still a large group of physicians who almost completely reject MC in the two dimensions described (i.e., process and treatment support). From a health economic point of view, it might be advisable not to focus politically (and also in the public discussion) on these “total rejecters.” The reason is that this could be economically inefficient compared to the solution outlined above for specific MC contracts that focus on only one dimension at a time. After all, the group of total objectors must first overcome the high hurdle of rejecting any form of MC before they would even engage in low‐threshold MC contracts.

With a view to future (quantitative) research on MC diffusion and acceptance in Germany, further studies on physicians' preferences should expand and concretize the MC options addressed here. In addition, the present work did not capture physicians' willingness to pay for MC options. The low number of cases could also be mentioned as a limitation with respect to this study's findings. Further research could, for example, investigate substitutional relations of preferences with respect to different MC elements in more detail. This would lead, for example, to a combination of the work of Rischatsch and Zweifei^7^ and the present approach. Furthermore, both the way MC is implemented and the relationships between diagnosis‐specific care and the respective preferences for MC are still open research areas. However, the very broad regional selection of physicians surveyed and the stability of the quantitative results in several statistical robustness checks (e.g., different rotation approaches and exclusion criteria in the factor analysis as well as different cluster solutions) suggest a high degree of stability and thus wide applicability of our results.

## AUTHOR CONTRIBUTIONS

**Dirk Oberschachtsiek**: Conceptualization; data curation; formal analysis; investigation; methodology; project administration; resources; software; supervision; validation; visualization; writing–original draft; writing–review and editing. **Andree Ehlert**: Conceptualization; data curation; formal analysis; investigation; methodology; project administration; software; supervision; validation; visualization; writing–original draft; writing – review and editing.

## CONFLICT OF INTEREST

The authors declare no conflict of interest.

## TRANSPARENCY STATEMENT

The lead author Dirk Oberschachtsiek, Andree Ehlert affirms that this manuscript is an honest, accurate, and transparent account of the study being reported; that no important aspects of the study have been omitted; and that any discrepancies from the study as planned (and, if relevant, registered) have been explained.

## Data Availability

The data that support the findings of this study are available from the corresponding author upon request.

## APPENDIX A

### A.1

**Table A1:** Factor loadings (after varimax rotation, factor loadings below 0.3 are suppressed)

Variable	Factor 1	Factor 2	Uniqueness
Documentation	**0.84**		0.22
Case management	0.57	0.56	0.36
Appointment coordination	**0.75**		0.35
Treatment organization		**0.69**	0.44
Specialist circles and meetings	0.45	**0.50**	0.55
Recourse defense	0.49	0.51	0.50
Administration	**0.73**	0.32	0.37

**Table A2:** Indicators for determining the number of clusters

	Duda/Hart		
Number of clusters	Je(2)/Je(1)	pseudo T‐squared	Calinski/Harabasz pseudo‐F
1	0.49	89.85	‐
2	0.51	57.59	89.85
3	0.39	42.62	96.81
4	0.65	22.31	92.74
5	0.46	36.36	93.32
6	0.44	20.23	102.55
7	0.28	30.94	106.12
